# The Pulp Stones: Morphological Analysis in Scanning Electron Microscopy and Spectroscopic Chemical Quantification

**DOI:** 10.3390/medicina58010005

**Published:** 2021-12-21

**Authors:** Aleksandra Palatyńska-Ulatowska, Marcos Cook Fernandes, Krystyna Pietrzycka, Agata Koprowicz, Leszek Klimek, Ronaldo Araújo Souza, Marieli Pradebon, José Antonio Poli de Figueiredo

**Affiliations:** 1 Department of Endodontics, Conservative Dentistry and Endodontics, Medical University of Lodz, 251 Pomorska Str., 92-213 Lodz, Poland; krystyna.pietrzycka@umed.lodz.pl (K.P.); agata.koprowicz@umed.lodz.pl (A.K.); 2 Department of Endodontics, Bahiana School of Medicine and Public Health, R. Silveira Martins, Salvador 41150-100, BA, Brazil; marcos.cook@hotmail.com (M.C.F.); ronaldosouza.endo@gmail.com (R.A.S.); 3 Institute of Materials Science and Technology, Technical University of Lodz, 1/15 Stefanowskiego Str., 90-924 Lodz, Poland; leszek.klimek@umed.lodz.pl; 4 Oral Histology and Endodontology Department, Federal University of Rio Grande do Sul, UFRGS, R. Sarmento Leite, n° 5000–Sala 134, Porto Alegre 90050-170, RS, Brazil; marielipradebon@hotmail.com (M.P.); poli.figueiredo@outlook.com (J.A.P.d.F.)

**Keywords:** dental pulp calcifications, denticles, endodontic treatment, pulp stones, pulp mineralized nodules

## Abstract

*Background and objectives:* Pulp stones are hard tissue structures formed in the pulp of permanent and deciduous teeth. Few studies have evaluated their morphology and chemical composition. However, their formation, composition, configuration and role played in overall health status are still unclear. Clinically, they may be symptomatic; technically, they impede access during endodontic therapy, increasing the risk of treatment errors. Thus, this study aimed to morphologically analyze pulp stones and present their chemical quantification, identifying their main chemical elements. It also correlates the results with their possible induction mechanisms. *Materials and Methods:* Seven pulp nodules were collected from molar teeth needing endodontic treatment. The morphology of the stones was analyzed by scanning electron microscopy (SEM), and their chemical composition was determined by X-ray dispersive energy spectroscopy (EDX). *Results:* These structures varied considerably in shape, size and topography. The site of the stones in the pulp cavity was the factor that most affected the morphology. The majority of the stones found in the pulp chambers presented nodular morphology, while those in the root canals presented a diffuse shape, resembling root canal anatomy. The topography of the nodules showed heterogeneous relief, revealing smooth and compact areas contrasting with the rugged and porous ones. The chemical composition varied depending on the location of the nodule in the pulp cavity and the relief of the analyzed area. Radicular stones presented considerably lower calcium and phosphorus content than coronary nodules. *Conclusions:* The high cellularity rate of the coronal pulp predisposes this region to nodular mineralizations around injured cells. The presence of larger caliber vascular bundles and higher collagen fiber content in radicular pulp determines a diffuse morphological pattern in this region. Understanding the morphology and chemical composition of the pulp stones allows future translational pathways towards the prevention or treatment of such conditions.

## 1. Introduction

The term “mineralization” is used when an inorganic substance precipitates in an organic matrix. Dental pulp mineralization means hard tissue structures formed along the pulp connective tissue of permanent and deciduous teeth. These structures are considered ectopic mineralizations and are routinely viewed in intraoral radiographs as radiopaque masses within the pulp cavity. The prevalence in the population is around 36% and molar teeth are the most frequently affected by this process [1,2,3].

The etiology of pulp mineralization is still a matter of debate in the literature. It is believed that long-standing and low-intensity stimulus on dental pulp tissue allows its initiation and development. Aging, traumatic occlusion, dental wear, orthodontic treatment, periodontal disease, chronic caries, deep restorations, as well as genetic and idiopathic factors are amongst conditions associated with pulp mineralizations [4,5,6].

There are several variations of pulp mineralization, either in structure, morphology, dimensions, or location [7]. Within the pulp chamber, they present a nodular format, generally spherical or ovoid. It sometimes mimics the internal anatomy of the coronal aspect of the pulp cavity. In the root canal space, they present a more diffuse structure, with tubular or cylindrical configuration, partially following the root canal design. The size varies from microscopic bodies to structures occupying the whole pulp chamber space [8].

Despite its general appearance microscopically, there is even more heterogeneity and complexity in pulp stones. Variations range from smooth and compact surfaces through porous and irregular, to crests and depressions [9].

The term “pulp nodule” was used to define mineralization that occurs within the connective tissue of the dental pulp. They were classified as true, false or diffuse. The true nodules are considered the ones that are composed of dentine, with dentinal tubules within and covered externally by odontoblasts. The false nodules are atubular, with concentric deposits of mineral tissue, incremented outwards of an initiating nucleus composed of organic matter. Diffuse nodules do not have definite patterns and occur dispersedly within the dental pulp, more often in radicular pulp, associated with blood capillaries and fibrillar structures [10,11].

Another classification for nodules when they are described as free, adherent, and embedded is in relation to dentin walls. Free nodules possess no connections with dentine, being confined to the dental pulp connective tissue. The adherent nodules are in intimacy with dentine walls, whilst embedded nodules are incorporated into dentinal mass during dentinogenesis. The chemical composition of these mineralizations is scarce in the literature. Some describe a predominance of calcium and phosphate in a similar proportion to hydroxyl apatite. Zinc, magnesium, sodium and copper were also reported [9,12].

Associations between pulp mineralizations and systemic conditions were made, especially in conditions that lead to ectopic mineralizations of other connective tissues, such as nephrolithiasis and atherosclerosis [13,14,15,16]. It appears that pathological mineralizations follow a similar pathway as the physiological process of hard tissues mineralization [17,18].

Clinical relevance of pulp mineralizations relates to technical difficulties in locating, accessing and preparing root canals [19]. These obstacles may result in complications such as zipping, ledging or perforations. Other authors suggest that mineralizations may compress nervous structures and elicit pain [20,21].

Considering the high occurrence of dental pulp mineralizations, little has been published as to their morphology, microscopic features and chemical composition. This could provide information about possible mechanisms leading to their formation and therefore provide a better understanding of their etiological factors. This study aims to assess morphology and chemical composition through SEM and EDX, correlating with possible mechanisms involved in their formation.

## 2. Materials and Methods

### 2.1. Study Design

**Figure 1:**
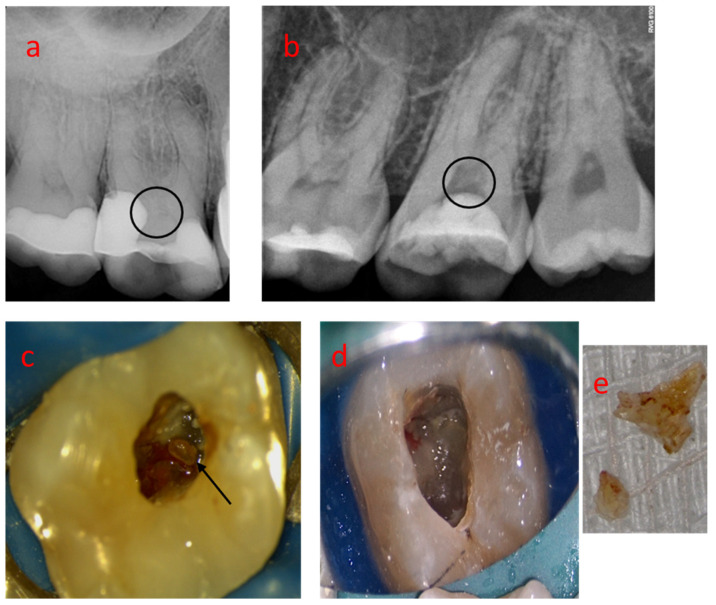
(**a**,**b**) Preoperative radiographs displaying dental pulp nodules within upper molars’ coronal pulp; (**c**) Pulp nodules visible during access (arrows); (**d**,**e**) A diffuse pulp nodule in a pulp cavity and after the removal.

This study comprises a project approved by the Ethical Committee of the Medical University of Lodz (RNN/413/19/KE 12.12.2019). Seven pulp nodules were collected from molar teeth needing endodontic treatment. The presence of the nodules was not a criterium for endodontic treatment. The reasons for it were pulp necrosis, inflammation, or restorative purposes. Samples of the nodules were gently removed during endodontic treatment provided by the Endodontic Department, Medical University of Lodz, Poland. Preoperative radiographs could display pulp mineralizations. Then, access cavities were planned to preserve these structures (Figure 1). All patients signed informed consent forms.

### 2.2. Sample Collection

A sample collection followed a structured clinical protocol. Following anesthesia (2% lidocaine 1:100,000) which was performed regardless of pulpal state (necrosis or inflammation), isolation was achieved with a rubber dam. Carious tissue was removed and restoration adjusted to allow careful access with the aid of a clinical microscope (Smart Optic, Seliga, Poland), which helped to maintain integrity of pulp mineralizations. Access was provided with high-speed rounded and tapered diamond burs under air–water spray cooling in order to avoid heat generated on the mineralized bodies. Irrigation consisted of 2.5% sodium hypochlorite, thoroughly used to dissolve vital or necrotic pulp tissue, and to remove dentine debris from mineralization surface.

When present in the pulp chamber, the free nodules were removed with the help of sharp curettes to smoothly dislocate and preserve the structural integrity (Figure 1). Some nodules adhered to the cavity walls and fracture could not be avoided. Others were fragmented because of their dimensions, which turned impossible to remove the whole structure in the cavity access. The Start-X ultrasonic tips (Dentsply, Switzerland), BUC #2 ultrasonic tip (Obtura Spartan, Algonquin, IL, USA) or Munce Discovery Burs (CJM Engineering Technologies, Ojai, CA, USA) were used in these cases.

**Figure 2:**
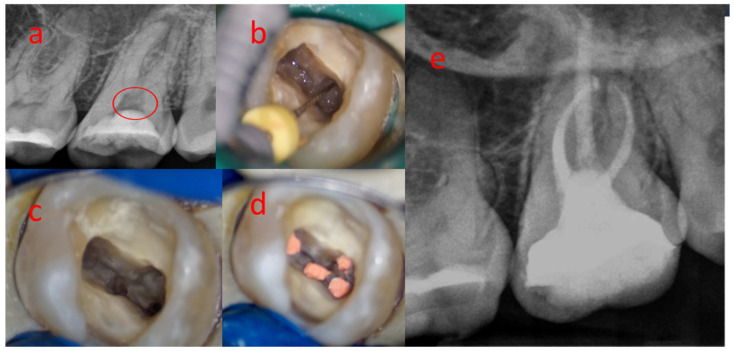
Removal of pulp mineralization followed by endodontic treatment: (**a**) Preoperative radiograph; (**b**) Identification of canal orifices; (**c**) The pulp chamber after the pulp stone removal; (**d**) Tooth after the endodontic treatment; (**e**) Postoperative radiograph.

Mineralizations located in the root canal were removed with a #08 or #10 K file, without filing action, just dislodging them with slow motions until they came out naturally or at the range of the sharp curette. After that, endodontic treatment followed a regular protocol for each tooth. (Figure 2).

### 2.3. SEM and EDX Analysis

After the collection, samples were kept in distilled water, renewed every 24 h. Then an ascending sequence of alcohol dehydrated the samples which were fixed in stubs and metalized with palladium gold. Images were obtained in the Philips XL 30 microscope, operating at 15 Kv, with magnifications varying from 30× to 50,000×. The whole surface of the mineralizations was searched in order to choose the most representative sample areas.

Chemical analysis of the samples was performed using X-ray dispersive energy spectroscopy (EDX). The considered areas were smooth and compact, as well as irregular and porous. They were exactly the same as the ones chosen for image acquisitions. This way there was correspondence between SEM images and the EDX quantification.

## 3. Results

Morphologic analysis of each sample was performed. The location, predominant topography and morphology are listed in Table 1.

### 3.1. Sample 01

**Figure 3:**
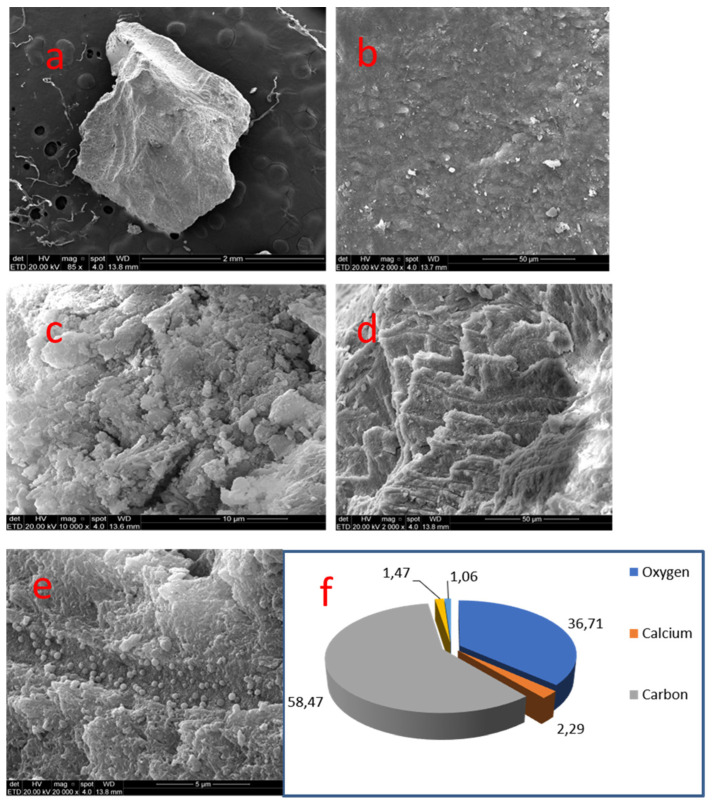
Sample 01: (**a**) Fragment of a coronal nodule (SEM, 85×); (**b**) Smooth and compact surface area (SEM, 2000×); (**c**) Irregular and porous area (SEM, 10,000×); (**d**) Tubular structures along fragmented area (SEM, 2000×); (**e**) Tubular structures containg calcospherites (SEM, 20,000×); (**f**) Results of chemical analysis of the sample with X-ray dispersive energy spectroscopy (EDX).

The sample consisted of a small fragment of a diffuse nodule from the pulp chamber of a lower molar, with an ovoid shape and irregular topography, sizing approximately 2 mm. There were areas in which the surface was smooth and compact and other porous areas that had an irregular relief with elongated and spherical structures. In a fragmented area there were tubular structures along the nodule, in a regular orientation, some of them parallel. Higher magnification showed a tubular structure of about 3 μm in diameter with calcospherites projected towards the tubule (Figure 3).

### 3.2. Sample 02

**Figure 4:**
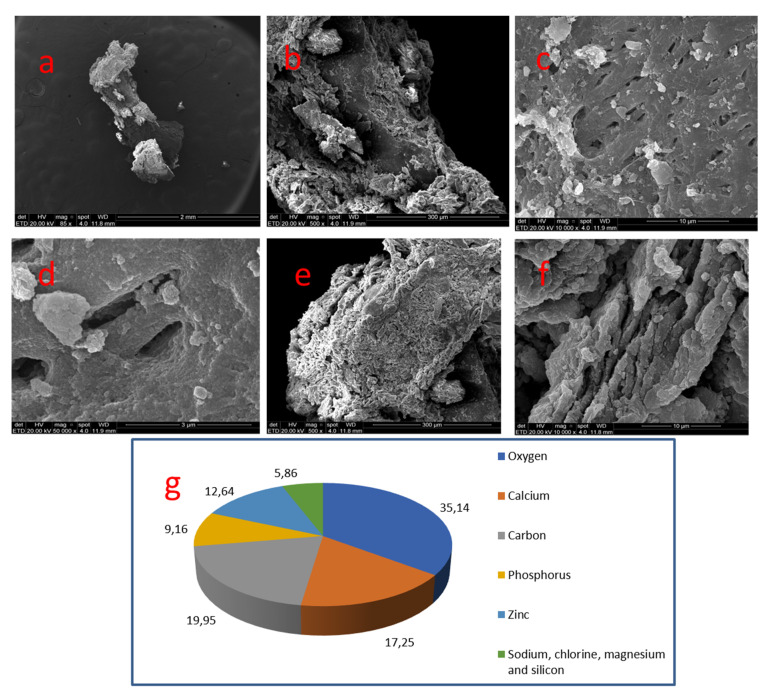
Sample 02: (**a**) irregular topography (SEM, 85×); (**b**) Irregular areas, with little areas of smooth and compact surface (arrow) (SEM, 500×); (**c**) Pores along the smooth surface (SEM, 10,000×); (**d**) Spherical crystalizing pattern (SEM, 50,000×); (**e**) Nodule pole with very irregular surface (SEM, 500×); (**f**) Crests and depressions (SEM, 10,000×); (**g**) Results of chemical analysis of the sample with X-ray dispersive energy spectroscopy (EDX).

This coronal mineralization of about 2.0 × 0.5 mm, in an elongated ovoid shape and very irregular topography, displayed a few areas of a smooth and compact surface. At 10,000× the smooth and compact surface showed pores of about 1.0 to 2.5 μm, resembling tubule openings. At higher magnification, a spherical crystalizing pattern could be seen. At the nodule poles, the surface was very irregular with crystal structures forming crests and depressions without a definite pattern (Figure 4).

### 3.3. Sample 03

**Figure 5:**
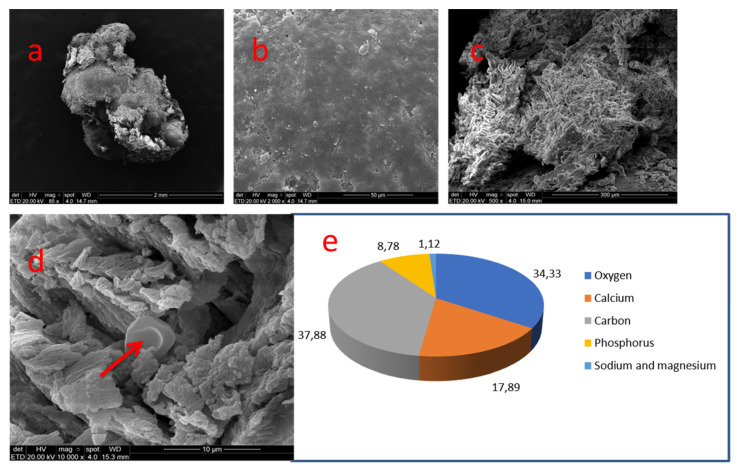
Sample 03: (**a**) Irregular topography of the coronal nodule (SEM, 85×); (**b**) Smooth and compact surface (SEM, 2000×); (**c**) porous zone and irregular topography (SEM-500×); (**d**) disorganized crystal formation. Presence of erythrocyte (arrow) (SEM, 10,000×); (**e**) Results of chemical analysis of the sample with X-ray dispersive energy spectroscopy (EDX).

At 85×, this coronal nodule of about 2.0 mm with ovoid format contained very irregular topography. At 2000× a small smooth and compact area could be seen. Another area of the nodule showed a porous surface and irregular relief with a disorganized crystalizing pattern. At higher magnification, an erythrocyte could be seen within a depression site (Figure 5).

### 3.4. Sample 04

**Figure 6:**
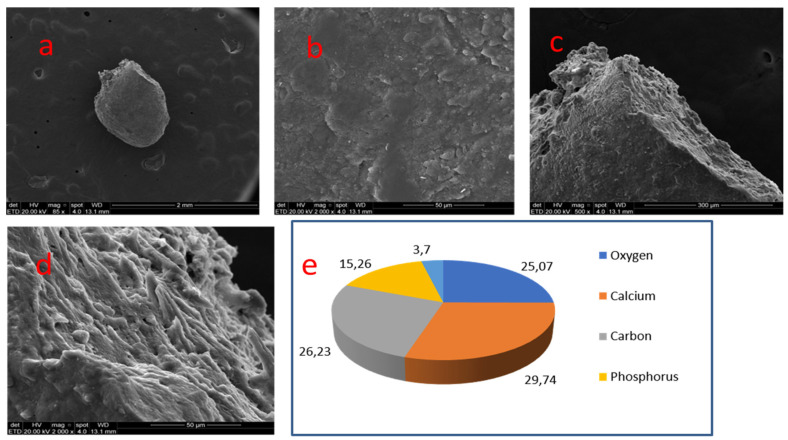
Sample 04: (**a**) An ovoid nodule with a smooth surface and compact presentation (SEM, 85×); (**a**,**b**) closer view of the smooth and compact surface (SEM, 2000×); (**c**) prominence with porous surface (SEM, 500×); (**d**) porous area with chip-like projections (SEM, 2000×); (**e**) Results of chemical analysis of the sample with X-ray dispersive energy spectroscopy (EDX).

A 1.0 mm compact and smooth ovoid nodule was removed from the coronal pulp. A prominent area could be seen along the nodule, with a more porous surface and irregular crystal pattern and chip-like projections (Figure 6).

### 3.5. Sample 05

**Figure 7:**
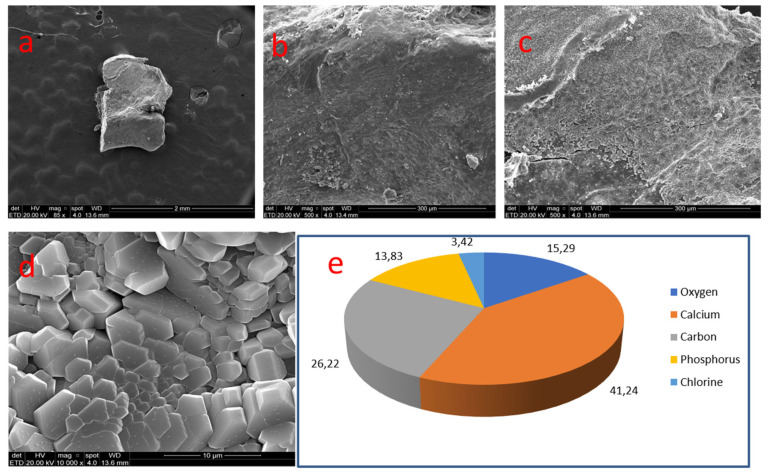
Sample 05: (**a**) Rectangular nodule (SEM, 85×); (**b**) Smooth and compact surface (SEM, 500×); (**c**) surface covered with exogenous substance (SEM, 500×); (**d**) sodium chloride crystals (SEM, 10,000×); (**e**) Results of chemical analysis of the sample with X-ray dispersive energy spectroscopy (EDX).

A 1.0 mm nodule, rectangular in shape, sizing 1.0 mm, was removed from the pulp chamber. It exhibited smooth and compact areas on most of the surface. A fracture zone was found at the left border of the structure. Certain areas of the nodule covered by an exogenous layer were observed at 500×. At 10,000× the sodium chloride crystals could be seen (Figure 7).

### 3.6. Sample 06

**Figure 8:**
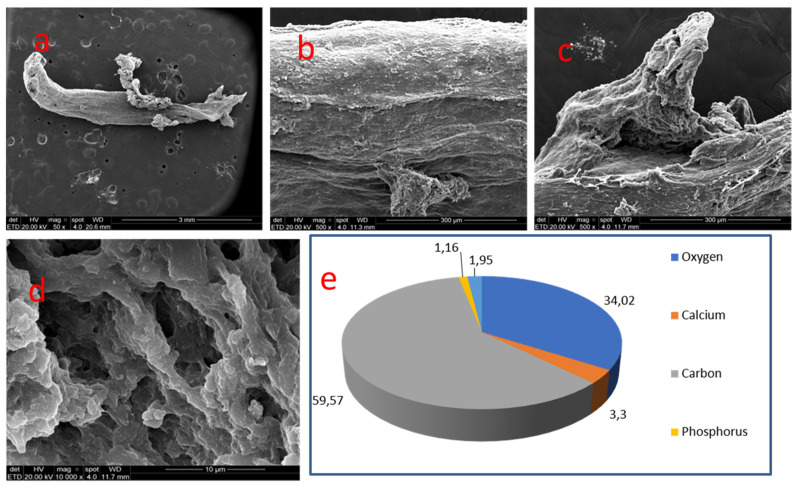
Sample 06: (**a**) radicular pulp mineralization with a tubular elongated pattern (SEM, 50×); (**b**) surface with sulcus and sinuous tracts (SEM, 500×); (**c**) Surface with projections and irregular relief (SEM, 500×); (**d**) crests and sulcus with deep pores (SEM, 10,000×); (**e**) Results of chemical analysis of the sample with X-ray dispersive energy spectroscopy (EDX).

This radicular diffuse mineralization presented a cylindrical elongated morphology, resembling 4 mm long root canal space. It was irregular, with sulcus and a sinuous pathway. Sometimes content was coiled and random. In other areas of the nodule surface, there were projections with irregular relief. The crystal formation pattern was spherical, sometimes elongated. Additionally, crests, sulcus and pores were apparently deeper than the ones found in pulp chamber nodules (Figure 8).

### 3.7. Sample 07

**Figure 9:**
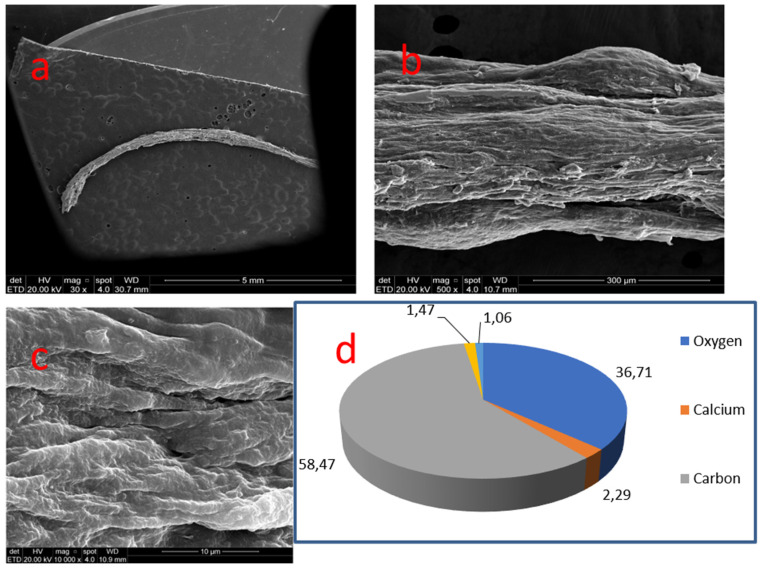
Sample 07: (**a**) Radicular pulp nodule with fillet-like morphology (SEM, 30×); (**b**) Surface with sulcus and sinuous tract (SEM, 500×); (**c**) Fusing coronal pattern with deep indentations (MEV, 10,000×); (**d**) Results of chemical analysis of the sample with X-ray dispersive energy spectroscopy (EDX).

This radicular pulp mineralization exhibited a diffuse elongated cylindrical fillet-like morphology, corresponding to the root canal space, 7.0 mm long. Irregular topography followed a sinuous tract with sulcus along the nodule. At 10,000×, topography denoted a crystal formation of fused cords with deep indentations (Figure 9).

### 3.8. EDX Chemical Quantification

Distinct mineralization areas were quantified in EDX, based on the sites chosen to verify the smooth and compact surfaces vs. those irregular and porous ones. It was noted that the site of election for EDX quantification influenced the predominance of certain elements.

Sample 01 contained smooth and porous areas. The composition was: oxygen (41.50%), calcium (24.93%), carbon (17.95%) and phosphate (11.58%). Traces of zinc, sodium, chloride and magnesium were also detected.

In sample 02, a porous area was selected. Oxygen (35.14%), carbon (19.95%) and calcium (17.25%) were the most frequently occurring elements. A great quantity of zinc was found (12.64%) and was bigger in amount than phosphate (9.16%). Traces of chloride, sodium, magnesium and silica were present.

In sample 03 a smooth and compact area comprised of carbon (37.88%) and oxygen (34.33%), followed by calcium (17.89%) and phosphate (8.78%). Traces of sodium and magnesium were detected.

In the smooth and compact area of sample 04, calcium (29.74%), carbon (26.23%), oxygen (25.07%) and phosphate (15.26%) were found. Zinc, sodium and magnesium were detected in minimal proportions.

There were two analyses carried out in sample 05. A smooth area presented calcium (41.24%), carbon (26.22%), oxygen (15.29%) and phosphate (13.83%). Traces of chloride were present. Another area revealed a great quantity of chloride (61.37%) and sodium (37.54%), with a minimal trace of calcium (1.09%).

Samples 06 and 07, located in radicular pulp, displayed carbon (59.57%/58.47%) and oxygen (34.02%/36.71%). Small amounts of calcium (3.30%/2.29%) and phosphate (1.16%/1.47%) could be detected, followed by traces of sodium, magnesium, chloride, sulfur and silica.

The presence of gold in samples was not taken into consideration, as this element is part of the sputtering process, needed for SEM.

## 4. Discussion

Ectopic mineralizations may occur in the proximity of pulp connective tissue. They are dystrophic calcifications caused by tissue alterations that predispose the deposit of calcium phosphate within the pulp extracellular matrix [22].

It is known that long-standing and low-intensity insults, such as trauma, chronic caries, deep restorations, chronic periodontal disease, occlusal load, and dental wear are amongst etiological factors for pulp mineralization [4,23,24]. They tend to break down pulp connective tissue homeostasis and provoke blood circulation disturbances, degenerative processes and small areas of pulp necrosis.

Alkaline phosphatase is pivotal in mineralization processes, though a cleavage of pyrophosphate and supplying phosphate for crystal nucleation. The enzymes annexins II, V and VI create calcium channels in cell vesicle membranes, allowing these ions to penetrate. These are the ways that calcium phosphate crystals are created [25].

Substance P also seems to contribute to the mineralization process. It is a neuropeptide released by neural terminations in the presence of a pathological stimulus, including caries, occlusal trauma and cavity preparations [26,27]. It regulates the process of apoptosis and is related to degenerative alterations that lead to ectopic mineralizations [28].

Hypoxia in pulp tissue seems to elicit dystrophic calcifications [29]. The aging of the pulp tissue is another factor that stimulates mineralization. In these cases, there is a reduction of pulp vascular, nervous and cellular supply, especially in the pulp chamber, resulting in hyaline degeneration, raising the fibrillar content, with condensed collagen [11,22,30,31]. Tranasi et al. [32] observed a raise in the expression of genes related to apoptosis in cells of aging pulps.

Several authors assume that ectopic mineralizations of connective tissues are similar to physiological processes of hard tissue mineralization [18,33,34]. Hard tissue formation cells, such as osteoblasts, odontoblasts and cementoblasts produce vesicles containing early-onset calcium phosphate that sprout from the plasmatic membrane to the extracellular matrix. These structures, known as matrix vesicles, create a microenvironment that is favorable to the initial enucleation of hydroxyl apatite crystals. The vesicle membranes contain proteins that regulate crystal formation, are alkaline phosphatase and annexins amongst them. Factors promoting and inhibiting mineralization actively regulate this process [35,36,37].

Ectopic mineralizations may occur in two distinct ways: 1. through the release of matrix vesicles that acquire osteogenic potential by a pathological stimulus; 2. through organic structures that are able to attract calcium phosphate salts, such as apoptotic and necrotic cells, small thrombus, and hyalinized extracellular matrix [38,39,40].

The morphological pattern of ectopic mineralizations within dental pulp connective tissue seems to be influenced by the location and extension of pulp tissue alterations. There are two main morphological patterns: nodular (either spherical or ovoid) and diffuse (without a definite pattern or reflecting pulp chamber or root canal space) [1].

SEM analyses in this study showed variations in shape, size and configuration of the dental pulp mineralized structures. Location within the pulp cavity influenced the morphology of the mineralizations the most. Nodules obtained from the pulp chamber tended to display an ovoid format, an exception to one that had a diffuse configuration. In the root canal space, mineralizations were all diffuse, cylindrical and elongated. The above stays in accordance with other studies, such as Foreman’s [8]. This author suggests that radicular mineralizations coalesce towards a dense mass. Le May and Kaqueler [41] observed that most nodules are round or oval, but they are able to produce more complex configurations.

Nodular mineralizations are more common in the pulp chamber [1,6]. This area contains more cellularity, together with well-distributed vessels and nerves and thin collagen fibers. Under the pathological stimulus, cells could undergo apoptosis and necrosis. Capillaries could tear down and a small thrombus with platelets could be seen. Small cell membrane fragments could release phosphatidylserine which combines with calcium and inorganic phosphate, initiating mineral accumulation and a more nodular configuration [20]. Apoptotic cells, if not phagocyted, speed up pathologic mineralization [33]. These cell remnants do not need matrix vesicles or mediation of alkaline phosphatase [34]. Therefore, a growth of radial increments of hydroxyl apatite crystal growth would facilitate spherical or ovoid structures [12].

Diffuse mineralizations are more frequent in radicular pulp [1,11]. This area contains fewer cells and more abundant collagen fibers, together with more robust vessels and nerves, following the root canal long axis. Circulatory alterations or chemical mediators released by oxidative stress could stimulate the differentiation of smooth muscle cells from dental pulp arterioles into cells with osteogenic potential. These metaplastic cells release matrix vesicles which spread through the basal membrane along the vessels and deposit early calcium phosphate crystals. Then mineralization will occur through heterogenic nucleation [18,39,42,43]. Vascular alterations mediated by substance P are a response to occlusal trauma and atherosclerotic alterations predispose the adventitious layer of pulp vessels to mineralization [44]. Since matrix vesicles are diffuse around tissue structures, it could be speculated that various sites with apatite crystals will deposit within the extracellular matrix, determining a diffuse mineralization pathway.

A greater fibrillar content of radicular pulp could lead to more extensive hyaline degeneration areas when subjected to a pathological stimulus or due to aging. The neural sheath could suffer lipidic degeneration, first at endoneurium, then perineurium, until all neural structure is affected. This serves as an organic scaffold to calcium phosphate crystal deposition [20,30]. Therefore, we believe extensive areas of tissue degeneration also tend to present a diffuse pattern, considering heterogenous nucleation which propagates randomly over an altered extracellular matrix, incorporating fibrillar, vascular and nervous structures.

One coronal nodule with a diffuse pattern was observed in the study. This finding is not uncommon when more extensive areas of tissue alteration such as hyaline degeneration in aging pulps are present. Macrophages have the potential of secreting matrix vesicles under the stimulus of proteins released by stressed cells [45].

There were two main topographic patterns of the mineralized structures: smooth and compact surfaces and irregular and porous and relief. Nodules collected in the pulp chamber showed heterogeneous sites, with smooth and compact areas, concomitantly with porous and irregular areas. This could represent distinct moments in the mineralization processes where smooth and compact zones are more mature and porous, whereas irregular relief gets more active in course of the mineralization process.

Once initiated, nodule formation occurs through heterogeneous nucleation and calcium and phosphate ions within the extracellular matrix are adsorbed by the crystalline structure being formed. Mineralization within connective tissues depends on the saturation of calcium and phosphate and on initiating and inhibiting factors. Non-collagenous proteins within the extracellular matrix play a role in crystal nucleation and growth. They contain amino acids that influence hydroxyl apatite crystal morphology [46].

Heterogeneity in nodule topography may suggest the simultaneous occurrence of mineralizations with distinct pathogeneses. Spherical and ovoid structures are likely to present an incremental pattern of mineralization and crystal deposition around their initiation nucleus and generally display a smooth a compact surface. Some of the nodules inspected in this study seemed to contain diffuse areas and reefs. This could indicate the fusion of two structures forming a single nodule with two distinct pathogeneses. In this study, radicular nodules also showed variations that suggest distinct chronological phases in the mineralization process.

The presence of tubular structures within pulp mineralizations served as a parameter used to distinguish between true and false nodules. True nodules are composed of dentine and covered by odontoblasts but are rarely present in microscopical studies. Some authors assume that they are more frequent at the apical third of the root canal, due to the rupture of Hertwig’s epithelial sheath during root formation, being engulfed by dentine during dentinogenesis. Most cases are considered false nodules, because of the atubular feature and a concentric mineralization pattern [1,10].

One limitation of this study was an inability to visualize the internal aspect of these structures. However, in nodule 01, parallel, tubular structures were found in an area of fracture that exposed the nodular structure internally. These tubules, about 3.0 μm in diameter, are compatible with the average diameter of dentinal tubules (0.9 to 3.0 μm). The presence of calcospherites inside the tubular structures could indicate an active site of mineralization. When assessing the pre-operative radiograph, the pulp chamber showed atresia, possibly with tertiary dentine. Clinically it was recognized as diffuse mineralization occupying the pulp chamber, adhering to the dentine wall. It seems that during the removal of the mineralized structure, a part of dentine tissue was also removed together with the nodule, which might explain the presence of tubular structures. Calcospherites could indicate the onset and progression of dentinal sclerosis as a response to low intensity and long-lasting stimulus to the tooth. Nodule 02 also displayed pores along its smooth surface, which could represent tubular structures. We may infer that, similarly to nodule 01, dentine and nodular structure have fused.

The chemical composition of the mineralizations under EDX showed a predominance of carbon, oxygen, calcium and phosphorus, suggesting there are organic and inorganic phases. The location of mineralizations along pulp space, as well as their topography, whether smooth or porous, interfered with the composition of the analyzed areas.

The presence of carbon and oxygen seems to be more related to the organic phase of the nodules, as these components are abundant in organic molecules that compose the extracellular matrix of connective tissues which are engulfed during mineralization. Under optical microscopy Hillmann and Geurtsen [11] observed the presence of various types of collagen fibers distributed in a concentric mode in coronal pulp nodules. In radicular diffuse mineralizations, they found apatite deposits parallel to various types of collagen fibers. Apart from collagen, Ninomiya et al. [47] identified other proteins, such as osteopontin, which are involved in the mineralization process.

The inorganic phase of the nodules, represented by calcium phosphate salts, are crystalline forms of biologic apatite [9,12]. The literature on the mineral phase of apatite that composes pulp mineralizations is controversial. The molar ratio between calcium and phosphorus determines the mineral phase. Pulp nodules may contain hydroxyl apatite (Ca/P: 1.67), amorphous calcium phosphate (Ca/P: 1.5), dehydrated mono-hydrogen calcium phosphate (Ca/P: 1.0), octa calcium phosphate (Ca/P: 1.33) and tricalcium phosphate (Ca/P: 1.5) [48]. In pathological ectopic mineralization, there may be alterations in hydroxyl apatite molecules. There may be components that are not a part of the stochiometric formulation of hydroxyl apatite, Ca_10_(PO_4_)_6_(OH)_2_. Phosphate can be partially substituted by carbon, resulting in carbonate hydroxyl apatite.

In this study, the molar ratio varied from 1.55 to 2.98, as shown in Table 2. Radicular nodule 07 showed the lower molar ratio of the samples, which corresponded to the amorphous calcium phosphate phase. Other nodules 02 and 04 showed a Ca/P ratio of 1.88 and 1.94, respectively, which is similar to what was found by Aoba et al. [49], a Ca/P of 1.86. The other nodules showed a Ca/P ratio over 2.0. Trowbridge et al. [50] discovered a Ca/P ratio of 2.11, and infrared spectroscopy revealed that the mineral phase was carbonated hydroxyl apatite. According to Béres et al. [12], most pulp nodules are composed of carbonated hydroxyl apatite, which could explain the great presence of carbon in the samples of the present study. In turn, Arys et al. [51] reported a nodule composed of bruxite.

Danilchenko et al. [52] analyzed the mineral component of cardiovascular mineral deposits. Under EDX, they found a Ca/P molar ratio varying from 2.04 to 2.95, which is superior to the average hydroxyl apatite ratio of 1.67. The authors believe that instead of phosphorus, crystalline phases containing CaO and CaCO_3_ could justify the findings. Besides, excess calcium could be concentrated at the crystal surfaces. According to Simpson [53], the Ca/P molar ratio can be superior to the theoretical 1.67 of hydroxyl apatite because the substitution of phosphate ions by carbonate ions results in a higher Ca/P molar ratio. This could explain the Ca/P molar ratio variations between 2.03 and 2.98, found in the samples of this study.

In coronal pulp mineralizations, the most frequent elements were oxygen, carbon and calcium. It indicates how great the organic phase in the samples was. In the smooth areas, there was a greater concentration of calcium, whereas in the porous areas it was oxygen. In a porous zone of nodule 02, zinc was found in a greater concentration than phosphate. Sodium, chloride, magnesium and silica were also found in considerable amounts. This means the topography of mineralization may influence the incorporation of other chemical elements in its structure. Apart from copper, Béres et al. [12] also found high levels of zinc in pulp nodules. The presence of these elements could be possible due to the presence of the antioxidant enzyme Cu/Zn-superoxide dismutase, secreted by odontoblasts in order to control oxidative stresses. However, according to the authors, the amount of this enzyme is too small to be detected by means of the above method. Therefore, an occurrence of zinc in evaluated samples may have a different source, which requires further inquiry.

Nodule 05 presented a zone containing only sodium chloride crystals adhered to the surface. This might be so due to the use of sodium hypochlorite as an irrigating substance during endodontic treatment.

Radicular mineralizations reveal a considerably higher concentration of carbon and oxygen when compared to coronal mineralizations. The finding may show that in the above cases we can find more robust organic structures, such as collagen fibrils and vascular and nervous bundles, which suffer diffuse mineralization, whereas in coronal pulp mineralizations the organic compounds are few.

The presented study contributed to the debate on the dental pulp stones, trying to consolidate clinical and basic elements of their onset and behavior. They are apparently similar in process to physiological mineralization events in other tissues and organs. Cells, such as osteoblasts, odontoblasts and cementoblasts, produce matrix vesicles containing early-onset calcium phosphate that sprout from the plasmatic membrane to the extracellular matrix. Their proteins, such as alkaline phosphatase and annexins, regulate crystal formation. Despite efforts towards regulating ectopic mineralizations with drugs, no treatment is currently available [54]. Studies on supplementing the diet with magnesium to prevent systemic mineralization may be an alternative, as magnesium replaces calcium and its excess is excreted in the urine. Magnesium phosphate complexes are more soluble compared to calcium phosphate, leading to less mineralization [55]. However, this is still a hypothesis needing further investigation. More needs to be clarified in relation to mineralizations, as well as pulp stones. Three-dimensional observations could enhance the knowledge of such structures and may be a step forward into the future.

## 5. Conclusions

Based on the findings of this study, we could conclude the following:

− Most of the mineralizations found in the pulp chambers presented nodular morphology, while those located in the root canals presented a diffuse shape, resembling the anatomy of the root canals.
− The topography of the nodules showed heterogeneous relief, revealing smooth and compact areas contrasting with the rugged and porous ones.
− The chemical composition of the mineralizations varied depending on the location of the nodule in the pulp cavity and the relief of the analyzed area. Root mineralizations presented considerably lower calcium and phosphorus content than coronal nodules.
− A careful study of the literature may lead to a conclusion that the high cellularity rate of the coronal pulp predisposes this region to nodular mineralizations around injured cells, whereas the presence of larger caliber vascular bundles and higher collagen fiber content in the root pulp determines a diffuse morphological pattern in this region.

## Figures and Tables

**Table 1:** Descriptive table indicating location, relationship with dentin wall, morphology, size and predominant topography of mineralizations.

Nº Nodule	Location	Dentin Wall Relationship	Morphology	Size	Predominant Topography
01	Coronal pulp	Free	Difuse (ovoid fragment)	2.00 mm (diameter)	Irregular
02	Coronal pulp	Adhered	Nodular (elongated ovoid)	2.00 × 0.5 mm	Irregular
03	Coronal pulp	Free	Nodular (ovoid)	2.00 mm (diameter)	Irregular
04	Coronal pulp	Undefined	Nodular (ovoid)	1.0 mm (diameter)	Smooth and compact
05	Coronal pulp	Undefined	Nodular (rectangular)	1.0 mm (diameter)	Smooth and compact
06	Root canal	Adhered	Cylindric (string)	4.0 mm (length)	Irregular grooved surface
07	Root canal	Free	Cylindric (fillet)	7.0 mm (length)	Irregular grooved surface

**Table 2:** Chemical composition of evaluated pulp stones dependent on their topography and location.

Nodule No	Age of the Patient	Location	Topography of the Analyzed Area	1	2	3	4	5	6	Ca/P Ratio
01	30	Coronal	Smooth and porous	O 41.5%	Ca 24.93%	C 17.95%	P 11.58%	Zn, Na, Cl and Mg 4.04%	-	2.15
02	44	Coronal	Smooth	O 35.14%	C 19.95%	Ca 17.25%	Zn 12.64%	P 9.16%	Na, Mg and Si 5.86%	1.88
03	36	Coronal	Smooth	C 37.88%	O 34.33%	Ca 17.89%	P 8.78%	Na and Mg 3.7%	-	2.03
04	45	Coronal	Smooth	Ca 29.74%	C 26.23%	O 25.07%	P 15.26%	Zn, Na and Mg 3.7%	-	1.94
05	60	Coronal	Smooth	Ca 41.24%	C 26.22%	O 15.29%	P 13.83%	Cl 3.42%	-	2.98
06	61	Radicular	Smooth	C 59.57%	O 34.02%	Ca 3.3%	Cl, Na, Mg, S and Si 1.95%	P 1.16%	-	2.84
07	24	Radicular	Porous	C 58.47%	O 36.71%	Ca 2.29%	P 1.47%	Cl, Na, Mg, S and Si 1.06%	-	1.55

## Data Availability

Data is contained within the article.
